# The first isolation of *Clostridium difficile* RT078/ST11 from pigs in China

**DOI:** 10.1371/journal.pone.0212965

**Published:** 2019-02-26

**Authors:** Li-Juan Zhang, Ling Yang, Xi-Xi Gu, Pin-Xian Chen, Jia-Li Fu, Hong-Xia Jiang

**Affiliations:** 1 National Risk Assessment laboratory for antimicrobial resistance of animal original bacteria, College of Veterinary Medicine, South China Agricultural University (SCAU), Guangzhou, China; 2 Guangdong Provincial Key Laboratory of Veterinary Pharmaceutics Development and Safety Evaluation, College of Veterinary Medicine, South China Agricultural University (SCAU), Guangzhou, China; University of Illinois at Chicago, UNITED STATES

## Abstract

We investigated the molecular characteristics and antimicrobial susceptibility of *Clostridium difficile* isolated from animals in China. We obtained 538 rectal swabs from pigs, chickens and ducks in 5 provinces during 2015 and 2016. *C*. *difficile* isolates were characterized by detection of toxin genes, multilocus sequence typing and ribotyping. And antimicrobial susceptibility testing was performed using the agar dilution method. Out of 538 samples, 44 (8.2%) were *C*. *difficile* positive with high prevalence in pigs (n = 31). Among these, 39 (88.6%) were toxigenic including 14 (31.8%) that were A^+^B^+^CDT^+^ and 13 (29.5%) A^+^B^+^. The remaining 12 (27.3%) were A^-^B^+^. We identified 7 ST types and 6 PCR ribotypes. The most predominant type was ST11/RT078 with toxin profile A^+^B^+^CDT^+^ and all were isolated from piglets with diarrhea. ST109 isolates possessed two different toxigenic profiles (A^-^B^-^CDT^-^ and A^-^B^+^CDT^-^) and although it was not the most prevalent sequence type, but it was widely distributed between chickens, ducks and pigs in the 5 provinces. All *C*. *difficile* isolates were fully susceptible to vancomycin, metronidazole, fidaxomicin, amoxicillin/clavulanate and meropenem but retained resistance to 4 or 5 of the remaining antibiotics, especially cefotaxime, tetracycline, ciprofloxacin, cefoxitin. The RT078/ST11 isolates were simultaneously resistant to cefotaxime, tetracycline, cefoxitin, ciprofloxacin and imipenem. This is the first report of the molecular epidemiology of *C*. *difficile* isolated from food animals in China. We identified the epidemic strain RT078/ST11 as the predominate isolate among the animals we screened in our study.

## Introduction

*Clostridium difficile* is a strictly anaerobic, spore-forming Gram-positive bacterium that colonizes the gastrointestinal tract of humans and animals and cause disease [1]. *C*. *difficile* pathogenesis is associated with the production of two enterotoxins (A and B) encoded by *tcdA* and *tcdB* on its pathogenicity locus. Some strains also produce a third toxin called binary toxin (cytolethal distending toxin, CDT) that is associated with increased disease severity and 30-day mortality [2].

*C*. *difficile* has emerged as the most common infectious cause of antibiotic-associated diarrhea and healthcare infections in developed countries. This is due to the emergence of hypervirulent strain, restriction endonuclease analysis type BI, North American pulsed field type 1 and PCR ribotype 027 (BI/NAP1/027) [1]. This strain produces toxins A, B and binary toxin CDT (A^+^B^+^CDT^+^) and possesses increased fluoroquinolone resistance and has been responsible for *C*. *difficile* infections (CDI) and outbreaks in North America, Canada and Europe since 2000 [3–6]. Currently, RT027 remains prevalent in North America and Europe,however, another PCR ribotype 078 is emerging as a significant human pathogen [7, 8].

The RT078 strain also produces toxins A, B and CDT (A^+^B^+^CDT^+^). Its importance as a human pathogen was first reported in the Netherlands and the incidence of infections caused by this strain increased from 3% to 13% between 2005 and 2008 [8]. At the same time, a similar increase with occasional outbreaks were recorded throughout Europe [4]. Recently *C*. *difficile* RT078 has increased to 4.4% of all *C*. *difficile* clinical isolates in North America [9]. Compared with RT027, this strain places lower-age populations at higher risk and is more frequently community associated than other strains [8]. Strain RT078 has been frequently isolated from livestock in Europe, United States, Canada, Australia, Japan and Taiwan suggesting that animals, particularly livestock, might be the reservoir for human CDI [10–12]. In addition, based on clonal relatedness of isolates derived from piglets in Europe, Taiwan and Japan, possible route of *C*. *difficile* RT078 transmission through piglets trading was suggested [13].

The exact evolutionary and epidemiological relationships between the *C*. *difficile* RT078 strains of humans and animals are still unknown due to the lack of discriminatory power of the current strain typing methods. Standard genotyping methods already highlight the genetic similarity between human and animal *C*. *difficile* RT078 strains and project an increase in zoonotic transmission [14–16]. More recently, whole-genome sequencing and core genome single-nucleotide polymorphism typing further confirmed this genetic overlap. This study indicated that asymptomatic farmers and their pigs can be colonized with clonal *C*. *difficile* RT078 [17].

Antibiotic resistance plays an important role in the spread of *C*. *difficile* strains and this is associated with the appearance of novel PCR ribotypes [5, 18]. The spread of the epidemic *C*. *difficile* RT027 and RT078 is mainly due to their fluoroquinolone resistance [5, 18, 19]. Previous studies have reported that the most common mechanism of fluoroquinolone resistance among *C*. *difficile* isolates are specific mutations in *gyrA* and *gyrB* [20, 21]. Recently, an increasing number of CDI studies in China have identified human-derived RT027 and RT078 strains [22–25]. Since no animal-related *C*. *difficile* strains have been reported, it is unclear whether these clones exist in animals in China, and the antimicrobial susceptibility of animal-derived strains. Therefore, we isolated *C*. *difficile* from fecal samples collected from different food animals in China to study the molecular epidemiology and antimicrobial resistance phenotypes of *C*. *difficile*, and further investigated the fluoroquinolone resistant determinants.

## Materials and methods

### Ethics statement

This study protocol was approved by the South China Agriculture University Animal ethics committee. The strains were isolated from cloacal swabs of pigs or chickens and ducks and the owners of the animals gave permission for their animals to be used in this study.

### Sample collection and *C*. *difficile* culture

During 2015 and 2016, we collected 538 stool samples from food animals from 5 Chinese provinces,which including 398 pig samples, 121 chicken samples and 19 duck rectal swabs. The samples were taken from five to ten animals per litter, and partial samples from all three reservoirs were sampled from the overlapping geographical locations.

Pig samples were taken from 4 different larger farms (total animal number >3000) and 2 small farms (total animal number<1000). Two of the 4 large farms were located in two different regions (Weifang and Qingdao) in Shandong, and the remaining 2 in Hubei and Guangdong. The 2 small farms were located in Henan and Jiangsu respectively. Those samples including 164 from nursery pigs (28–40 days old) in Jiangsu (n = 20) and Hubei (n = 144), 105 from piglets (14–20 days old) with diarrhea in Guangdong, 17 from sows (about 200 days old) in Henan and 112 from pregnant sows (>40 weeks old) from Weifang (n = 62) and Qingdao (n = 50).

We also collected 46, 47 and 28 adult chicken (about 28–40 days old) samples from 3 large-scale farms in Hubei, Shandong and Jiangsu, respectively. They were all healthy and asymptomatic. In addition, 19 rectal swabs from healthy 45 days old ducks were randomly collected from one farm in Shandong. All swabs were cryopreserved after collection and delivered rapidly to the laboratory.

Stool samples were incubated in *C*. *difficile* moxalactam-norfloxacin (CDMN, Oxoid, Cambridge, UK) broth supplemented with 0.1% sodium taurocholate at 37°C for 7 days. The culture samples were treated with alcohol to a final concentration of 75% at room temperature for 60 min before the anaerobic isolation of *C*. *difficile*. Then samples were centrifuged and the supernatant was discarded and the sediment was incubated in *C*. *difficile* moxalactam-norfloxacin selective medium (CDMN, Oxoid, Thermo-Fisher, Pittsburg, PA USA) at 37°C for 48 h. Presumptive *C*. *difficile* colonies were identified by matrix-assisted laser desorption/ionization–time of flight (MALDI-TOF) mass spectrometry (Shimadzu-Biotech) using instructions provided by the manufacturer. Strains were stored in brain heart infusion broth containing 20% glycerol at -80°C.

### Toxin gene profiling and molecular typing

Genomic DNA was isolated from purified colonies of *C*. *difficile* grown on the selective medium CDMN containing cysteine hydrochloride, norfloxacin and moxalactam for 48h 37°C under anaerobic conditions. Colonies were suspended in 300 μL water and DNA was extracted using a TIANamp Bacterial DNA Kit (Tiangen Biotech, Beijing, China) according to the manufacturer’s instructions. DNA samples were stored at -20°C.

PCR amplification of *tcdA*, *B*, *cdtA/B* and fragments of *gyrA* and gyr*B* gene were carried out as previously described [21, 26–28]. PCR products were analyzed by electrophoresis through 1.5% agarose. Fragments of *gyrA* and *gyrB* gene were directly sequenced, and the results were compared with the genome of *C*. *difficile* strain 630 (Accession number: NC_009089.1).

Multilocus sequence typing (MLST) was performed on all isolates as described previously using the following gene targets: *adk*, *atpA*, *dxr*, *glyA*, *recA*, *soda* and *tpi* [29]. All amplified products were commercially sequenced (Invitrogen, Shanghai China) and DNA sequences were submitted to the MLST database (http://pubmlst.org/*clostridium difficile*) to obtain the sequence type (ST).

PCR ribotyping (PR) was performed based on a previously published protocol [30]. The PCR products were separated by electrophoresis in 3% agarose gels for 2 h at 100 V and the PR profiles were analyzed using a CHEF-MAPPER System (Bio-Rad Laboratories, Hercules, CA, USA) to construct a dendrogram. *C*. *difficile* RT078 strain was donated by Professor Haihui Huang of Fudan University.

### Antimicrobial susceptibility testing

Antimicrobial susceptibility testing of all isolates was performed using the agar dilution method according to Clinical and Laboratory Standards Institute guidelines (CLSI) [31]. *Bacteroides fragilis* ATCC25285 and *C*. *difficile* ATCC700057 were used as quality control samples. The following 17 antibiotics were tested: ceftiofur (CTF), ciprofloxacin (CIP), cefoxitin (CXT), fidaxomicin (FDX), metronidazole (MTZ), vancomycin (VAN), clindamycin (CLI), tetracycline (TET), imipenem (IPM), meropenem (MEM), cefotaxime (CTX), erythromycin (ERY), ampicillin (AMP), chloramphenicol (CHL), amoxicillin-clavulanic acid (AMC) and moxifloxacin (MXF), fosfomycin (FOS). Interpretation of antimicrobial susceptibility was based on CLSI guidelines [31] and the European Committee on Antimicrobial Susceptibility Testing (EUCAST) [32]. For antimicrobial agents where no standard breakpoints were available from CLSI or EUCAST, resistance was considered as follows: ciprofloxacin, ≥8 mg/L; erythromycin, ≥128mg/L [33].

### Statistical analysis

MIC_50/90_ were carried out using SPSS software (Version 18.0).

## Results

### Strain isolation and toxin gene identification

We isolated 31 *C*. *difficile* strains from 398 pig samples at a rate of 7.8% and these strains possessed four different toxin type combinations. These included 19 from diarrhea piglets (Guangdong) and included 14 A^+^B^+^CDT^+^ and 5 nontoxigenic strains. The 9 isolates from pregnant sows (Shandong) were all (A^+^B^+^). The 2 strains from Hubei nursery pigs and the single Henan sow isolate were A^-^B^+^.

The chicken isolates included 9 (7.4%) positive for *C*. *difficile* including 6 from Hubei, 2 from Shandong and 1 from Jiangsu. The 4 isolates from Hubei and the single from Jiangsu were all A^-^B^+^ and the other 2 strains from Hubei along with 2 isolates from Shandong were A^+^B^+^. We also isolated 4 strains of *C*. *difficile* from 19 duck samples and all were A^-^B^+^ (Table 1).

**Table 1:** Genotypes and antibiotic resistance of *C*. *difficile* strains isolated from animal feces.

Toxin profile	Total No. of Strains	Multidrug-Resistant Patterns			Amino acid substitutions		ST^≠^	RT ^§^	Animal source
		Quadruple Drug Resistance	Quintuple Drug Resistance	Sextuple Drug Resistance	*GyrA*	*GyrB*			
A^-^, B^-^,	5	CIP/CXT/TET/CTX	CIP/CXT/TET/CTX/ERY	CIP/CXT/TET/CTX/ERY/MXF	Thr87-Ile	Ser366-Ala	109	GZ1	Pig
	1			CIP/CXT/TET/CTX/ERY/MXF			238	GZ1	pig
A^-^, B^+^,		CXT/CLI/CTX/ERY		CIP/CXT/TET/CTX/ERY/CLI			48	GZ6	Pig Chicken
	7	CIP/CXT/TET/CTX					240	GZ5	Duck
	4		CIP/CXT/TET/CTX/MXF			Ser366-Ala	109	GZ1	Chicken Pig Duck
A^+^, B^+^,	4	CIP/CXT/TET/CTX			Thr82-Ile		3	GZ4	Chicken
	9	CIP/CXT/TET/CTX	CIP/CXT/TET/CTX/IPM	CIP/CXT/TET/CTX/IPM/ERY	Thr82-Ile		35	GZ3	Pig
A^+^,B^+^,CDT^+^	14	CIP/CXT/TET/CTX	CIP/CXT/TET/CTX/IPM			Ser366-Val Ser416-Ala	11	GZ2(RT078)	Pig

≠Sequence Type.

§ Ribotype.

CIP, ciprofloxacin; CXT, cefoxitin; TET, tetracycline; CTX, cefotaxime; ERY, erythromycin; CLI, clindamycin; MXF, moxifloxacin; IPM, imipenem.

### Multilocus sequence typing (MLST) and PCR ribotypes

The 44 total *C*. *difficile* isolates possessed 7 MLST genotypes with sequence type (ST) 11 (14/44) as the most frequent followed by ST109 and ST35 with 9 members each. There were no new ST types represented in this group. However, ST11, ST35 and ST238 were only present in pig samples, ST3 were all derived from chicken samples and ST240 was present only in ducks. ST48 was present both in pigs and in chickens and interestingly, ST109 was present in all three animal groups (Table 1).

**Fig 1:**
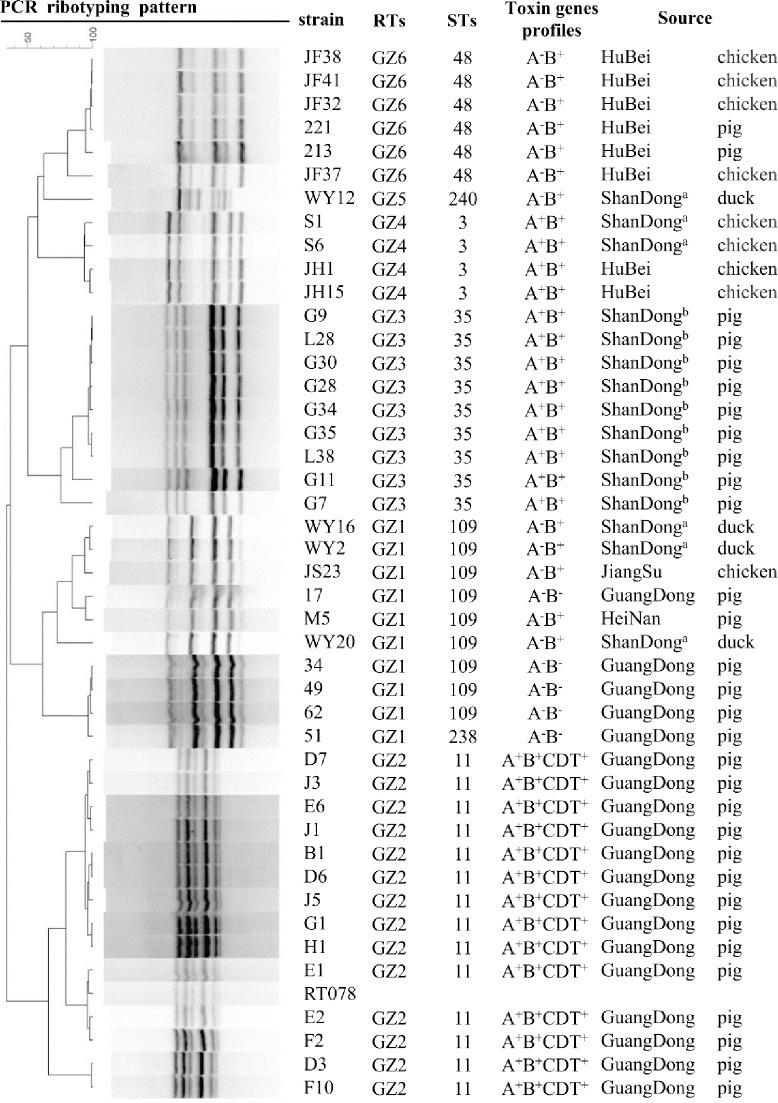
Cluster analyses based on PCR ribotyping of 44 *C*. *difficile* isolates. ^a^ Weifang, ^b^ Qingdao.

We also identified 6 different PCR ribotypes. However, due to a lack of strains for standardization, these were given the sequential names GZ1, GZ2, GZ3, GZ4, GZ5 and GZ6. All GZ2 strains were confirmed to be RT078 and all were isolated from piglets with diarrhea in Guangdong (Fig 1).

### Antimicrobial susceptibility and *gyrA*, and *gyrB* mutations

All our *C*. *difficile* isolates were fully susceptible to vancomycin, metronidazole, fidaxomicin, amoxicillin/clavulanate and meropenem, but showed different degrees of resistance to other antibiotics tested in this study (Table 2). Most of the strains were resistant to three or four antibiotics and the most frequent multidrug profile was CIP/CXT/TET/CTX. The strain of the predominant type RT078 (GZ2) we found in this study was resistant to IMP and possessed the most prevalent multi-resistance profile (Table 1).

**Table 2:** Minimum inhibitory concentrations (MICs) for 17 antimicrobial agents against 44 *C*. *difficile* animal isolates.

Antimicrobial agent	Resistance breakpoint	MIC_50_ (mg/L)	MIC_90_ (mg/L)	Range (mg/L)	Resistance (%)
Vancomycin	≥4^b^	0.5	0.5	0.03–1	0
Fosfomycin	-	8	16	2–128	-
Metronidazole	≥2^b^	0.06	0.25	0.03–0.25	0
Fidaxomicin	-	0.125	0.125	0.03–0.25	-
Clindamycin	≥8^a^	0.015	0.25	0.015->256	13.6
Amoxicillin-clavulanic acid	≥16^a^	4	8	0.125–128	2.3
Chloramphenicol	≥32^a^	4	8	0.06–32	4.5
Moxifloxacin	≥4^b^	2	32	0.125–32	29.5
Cefoxitin	≥64^a^	64	128	16–128	97.7
Imipenem	≥16^a^	8	16	0.06–64	36.3
Erythromycin	≥128^c^	64	128	1->512	45.5
Ciprofloxacin	≥8^c^	16	64	<0.015–64	93.2
Meropenem	≥16^a^	2	2	0.03–2	0
Ampicillin	≥2^a^	1	2	0.5–4	16
Tetracycline	≥16^a^	16	64	0.015–128	77.3
Cefotaxime	64^a^	64	64	0.03->512	95.5
Ceftiofur	-	64	128	0.015->512	-

MIC_50/90_, minimum inhibitory concentration for 50% and 90% of the isolates, respectively.

^a^ MIC breakpoints for *C*. *difficile* recommended by the Clinical and Laboratory Standards Institute [31].

^b^ MIC breakpoint was based on the recommendation by the European Committee on Antimicrobial Susceptibility Testing [32].

^c^ MIC breakpoints were calculated as previously reported [33].

We also observed high rates of fluoroquinolone resistance. The resistance rates to CIP were 93% (41/44) and 29% of these were also resistant to MXF. We also identified *gyrA* and *gyrB* amplicons for all isolates and 73% (32/44) of these carried mutations in either *GyrA* or *GyrB*. The 5 A^-^B^-^ pig isolates were resistant to both MXF (MIC range 16 to 32 mg/L) and CIP (MIC range 32 to 64 mg/L) and both possessed *GyrA* (T87I) and *GyrB* (S366A) mutations.

The 8 A^+^B^+^ strains from different sources were resistant to MXF (MIC range 16 to 32 mg/L) and CIP (MIC range 16 to 64 mg/L) and possessed T82I in the *GyrA*. The 14 A^+^B^+^CDT^+^ strains isolated from piglets were resistant only to ciprofloxacin (MIC = 16 mg/L) and possessed two *GyrB* mutations (S366V, S416A) and none in *GyrA*. The 5 A^-^B^+^ strains from different origins were only resistant to CIP (MIC range 16 to 64 mg/L) and possessed a single *GyrB* (S366A) mutation (Table 1).

## Discussion

In the present study, we isolated 31 *C*. *difficile* strains from pigs at a rate of 7.8%, which was significantly lower than previously reported rates of 36% ~ 50% [34, 35]. This difference can be accounted for by the animal ages since the prevalence of *C*. *difficile* in pigs decreases with age [36]. Our study samples were derived from nursery pigs and sows with only a few samples from piglets. Interestingly, strain RT078 (GZ2/ST11) predominated and these samples were all obtained from piglets. In addition, we found 9 *C*. *difficile* in chickens and 4 *C*. *difficile* strains from ducks with isolate rates at 7.4% (9/121) and 21% (4/19) respectively. Since China is the largest producer of chickens and ducks for food, the isolation of *C*. *difficile* from these animals indicates a potential public health threat.

Among the 44 *C*. *difficile* strains, 39 were toxigenic and the most common toxin genes profile was A^+^B^+^CDT^+^. These accounted for 36% (14/44) of the total and all were identified in isolates of ST11/RT078. It is remarkable that the predominant strain we found was the epidemic strain RT078, which were all isolated from Guangdong. Strain RT027 and RT078 strains have acquired unique mechanisms to metabolize low concentrations of the disaccharide trehalose, and this ability correlates with disease severity [37]. The addition of trehalose to animal feeds in China is not a common practice and was not used on the farms we examined in this study. Therefore, it is unlikely that the predominance of the RT078 strain in Guangdong is related to trehalose.

In humans, RT078 is one of the ten most frequently identified ribotypes and accounts for 4% to 8% of *C*. *difficile* clinical isolates in North America and Europe [4, 9]. In China, two reports have identified RT078 strains isolated from environmental surfaces and clinical patients in Zhejiang and Beijing [23, 25], our study is the first to report of RT078 isolation from piglets. *C*. *difficile* RT078 exists in a clonal population that often moves between livestock and human hosts independent of geographic barriers [38]. Although RT078 isolates have not been reported from humans in Guangdong, the identification of RT078 from piglets suggests a potential for zoonotic CDI risks in China in the near future.

All RT078 strains obtained from this study were fully susceptible to VAN, MTZ, FDX, CLI, AMC, CHL, MEN, ERY, AMP and CTF, but co-resistant to CXT, TET, CIP, CTX and IPM. Swine RT078 isolates reported as resistant to MXF showed the same *GyrA* mutation, T82I [10, 39]. However, all RT078 isolates in our study were susceptible to MXF and possessed two *GyrB* mutations (S366V, S416A). Interestingly, we found a relatively high resistance rate of 93% (13/14) to imipenem among the RT078 strains, while the overall IMP resistance rate of *C*. *difficile* isolated from Chinese medical clinics is very low [40]. A comparison of human and piglet *C*. *difficile* RT078 strains show that imipenem was the only difference in the antimicrobial resistance spectrum between human and pig isolates, with resistant rates at 29 and 50%, respectively [16]. This was unexpected since imipenem is not used in the swine husbandry.

Another important genotype was the A^-^B^+^ strain. We found that 31% (12/39) of the A^-^B^+^ strains were obtained from three animal types and distributed over five provinces. The A^-^B^+^ strain is the most prevalent strain obtained from clinics in China and it may also be the most prevalent strain from food animals in China [41, 42]. The strains we identified contained 3 different ST types (ST48, ST240 and ST109). It is worth noting that ST109 was not the dominant ST type, but possessed the widest geographic and animal host distribution. This suggests clonal transmission between different animals among different regions of China. We also identified 5 nontoxigenic strains belonging to ST109. Compared with the toxigenic ST109 isolates, these nontoxigenic ST109 strain were resistant to MXF and CIP and possessed both *GyrA* (T87I) and *GyrB* (S366A) mutations. In contrast, the toxigenic strains that were resistant to moxifloxacin and ciprofloxacin possessed single *GyrA* (T82I) mutations.

The remaining toxigenic strains were A^+^B^+^ strains that accounted for 33% (13/39) and possessed two STs; ST3 (4/13) and ST35 (9/13). The ST3 strains were isolated from chicken stools while ST35 strains were from pigs. All were CIP resistant and possessed the *GyrA* mutation T82I. It is worth noting that ST3 and ST35 are also common in human *C*. *difficile* isolates in China and ST3 was the first dominant strain [22]. This indicates that animals may be potential reservoirs of *C*. *difficile*.

In addition, we tested all *C*. *difficile* isolates for their minimal inhibitory concentration against 17 antimicrobial agents and the results showed that they were serious resistance to CIP, CTX, CXT and TET, with resistance rate at 93.2%, 95.5%, 97.9% and 77.3% respectively. Data from 30 studies published from 2012 to 2015 indicate that *C*. *difficile* clinical isolates are very commonly resistant to CLI, CXT, CIP, with resistance rate at 55%, 79%, and 99% [18]. The rate of antibiotic resistance varies widely among studies and may depend on geographic regions and local or national antibiotic policies, as well as the source of the sample (animal and human).

The high resistance rate of CIP (a second-generation FQ) in *C*. *difficile* was commonly observed in both this and other studies [18, 22].The fluoroquinolone resistance in Gram-negative bacteria is primarily the result of mutations in the chromosomal gene encoding the quinolone targets *gyrA*, *gyrB*, *parC* and *parE*. Single mutations in the *gyrA* gene confers low level quinolone resistance while high-level fluoroquinolone resistance from *gyrA* mutations often requires an additional single or double mutation in *parB* or *parC* [43, 44]. However, decreased susceptibility to fluoroquinolones in *C*. *difficile* is associated with the occurrence of a *gyrA* or *gyrB* mutation [10, 21]. Interestingly, human clinical isolates of *C*. *difficile* often exhibit Asp to Asn or Val mutations in 426 of *GyrB* [40]. Our research found that the primary mutations in *GyrB* is Ser to Ala. The *C*. *difficile* mutation in *GyrB* from Ser to Ala has also been reported in human clinical isolates from different countries [18, 20, 40]. Previous research indicated that the substitutions found in *GyrB* characteristic of certain *C*. *difficile* types and countries [20]. In addition, it is noteworthy that we found that A^-^B^-^ strains and A^+^B^+^ strains that showed the same quinolone resistance phenotypes (both resistant to CIP and MXF at similar levels), but they possessed different *gyrA* and *gyrB* mutations. Moreover, the A^-^B^+^ and A^+^B^+^CDT^+^ strains also carried different mutations in *gyrA* or *gyrB*, even though they possessed similar CIP resistance phenotypes (MIC range 16 to 64 mg/L). This suggests that the *gyrA* and *gyrB* mutation sites in *C*. *difficile* are not only related to fluoroquinolone resistance but also to toxin production ability.

## Conclusions

The present study is the first report of hypervirulent strain RT078/ST11 strain from piglets in China. This strain was multiply resistant to CXT/TET/CIP/CTX/IPM. Our results indicated that ST109 was the most widely distributed type of *C*. *difficile* from food animals in China. These ST isolates were obtained from different animals and provinces and possessed different resistance phenotypes. This study provides an important baseline for ongoing long-term surveillance of antimicrobial resistance and prospective tracking of prominent and emerging strain types in China.
